# Supplementary material for: Chromothripsis during telomere crisis is independent of NHEJ, and consistent with a replicative origin
Source: Genome Res. 2019 May;29(5):737–49. doi: 10.1101/gr.240705.118 (PMC6499312; doi:10.1101/gr.240705.118)
Supplement: Supplemental Material [file supp_gr.240705.118_Supplemental_file_1.zip › contigs/annotated_contigs/DB98/contig.2.DB98_length_537_mean_cov_8.14711359404.docx]

**DB98_length_537_mean_cov_8.14711359404**

```
ACGAAAAAAAAATTAGCCAGGTGTGGTGGCAGGCGCCTGTAGTCCCAGCTACTAGGGAGGCTGAGCCAGGAGAATGGTGTGAACCCGGG
 >chr7:139071073-139071329 - E=1e-128
AGGCGGAGCTTGCAGTAAGCCAAGATCACACCACTGCACTCCAGCCTGGACGACAGAGTGAGACTCCATCTCAAAAAAAAAAAATTTCC

AGTGACAGAGAAGAATATTTCCAGTGACAGAGATTTTGTAATTCTTTGTAAATGTGCTTGATGTTTGTGG|TT|TTGTTTCTATTATAA
                                                                       >chr7:139192338-
AACAACAAAAATAAGCATGGATTTTCTTATTGTAAAACTAACACGTAGGCTGTTTTCATGTAAACAACATATAAATGTTTAAAATAATT
139192627 - E=3e-156
TTTCTTTAGAATTTGGCATACTAATTACATGCGTCTAAGTACTTTTTAAATGTATGCAAAATAAATCCTTATGAACTTTTTTTCACACA

AATGGGATGACTTCATGCACTCTGCAAACTTGCTTCTTGTGTCATTTCATATAGCTCCATCACATTCTTAACCTAGAATTCCAGAGCAG

GCCAG
```
